# To Construct an Engineered (*S*)-Equol Resistant *E. coli* for *in Vitro* (*S*)-Equol Production

**DOI:** 10.3389/fmicb.2018.01182

**Published:** 2018-06-04

**Authors:** Hailiang Li, Shaoming Mao, Huahai Chen, Liying Zhu, Wei Liu, Xin Wang, Yeshi Yin

**Affiliations:** ^1^Key Laboratory of Comprehensive Utilization of Advantage Plants Resources in Hunan South, College of Chemistry and Bioengineering, Hunan University of Science and Engineering, Yongzhou, China; ^2^State Key Laboratory Breeding Base for Zhejiang Sustainable Pest and Disease Control, Institute of Plant Protection and Microbiology, Zhejiang Academy of Agricultural Sciences, Hangzhou, China; ^3^Hunan Provincial Key Laboratory for Forestry Biotechnology, College of Life Science and Technology, Central South University of Forestry and Technology, Changsha, China

**Keywords:** (*S*)-equol production, (*S*)-equol resistance, soybean isoflavone, transposon mutagenesis, *ydiS* gene

## Abstract

(*S*)-equol is one of the major metabolites of daidzein that is produced by human and animal gut bacteria. Most of the physiological functions of soybean isoflavones, such as anti-oxidative activity, anti-cancer activity, and cardiovascular protection have been ascribed to (*S*)-equol. However, only 30–50% people contain this kind of equol-producing bacteria, and therefore are able to convert daidzein to (*S*)-equol. Administration of (*S*)-equol may be more beneficial than soybean isoflavones. The aim of this study was to construct an engineered (*S*)-equol resistant *Escherichia coli* to enhance (*S*)-equol production *in vitro*. First, transposon mutagenesis libraries were constructed and screened to isolate the (*S*)-equol resistant mutant *E. coli* strain BL21 (*ydiS*) in order to overcome the inhibitory effects of (*S*)-equol on bacterial growth. Bacterial full genome scan sequencing and *in vitro* overexpression results revealed that the *ydiS* gene was responsible for this resistance. Second, the (*S*)-equol-producing genes L-*dznr*, L-*ddrc*, L-*dhdr*, and L-*thdr* of *Lactococcus* strain 20–92 were synthesized and cloned into compatible vectors, pETDuet-1 and pCDFDuet-1. These plasmids were subsequently transformed into BL21 (DE3) and its mutant BL21 (*ydiS*). Both engineered BL21 (DE3) and BL21 (*ydiS*) could use daidzein as substrate to produce (*S*)-equol under both anaerobic and aerobic conditions. As expected, engineered BL21 (*ydiS*) had faster growth rates than BL21 (DE3) when supplemented with high concentrations of (*S*)-equol. The yield and the daidzein utilization ratio were higher for engineered BL21 (*ydiS*). Interestingly, engineered BL21 (*ydiS*) was able to convert daidzein to (*S*)-equol efficiently under aerobic conditions, providing a convenient method for (*S*)-equol production *in vitro*. In addition, a two-step method was developed to produce (*S*)-equol using daidzin as substrate.

## Introduction

Soy isoflavones have multiple health benefits due to their anti-carcinogenic, anti-oxidant, and anti-atherosclerotic properties (Xiao et al., 2017). These chemicals also interact with the estrogen receptor, enabling them to act as weak to moderate phytoestrogens (Nielsen and Williamson, 2007). Interestingly, a variety of studies have suggested that the clinical effectiveness of isoflavones might be due to their metabolites (Setchell et al., 2002; Sarkar and Li, 2003; Cobb et al., 2006; Cooke, 2006; Jackman et al., 2007). In 2002, Setchell et al. (2002) proposed the “Equol Hypothesis," which posits that daidzein is converted to (*S*)-equol by gut bacteria in certain individuals, and that it is the equol that accounts for the noted health benefits of soy isoflavones.

Equol (7-hydroxy-3-[4-hydroxyphenyl]-chroman) was first isolated from equine urine in 1932 (Marrian and Haslewood, 1932) and was also identified 50 years later in human urine as a metabolite of soy isoflavones (Axelson et al., 1982). Equol is not present in soybeans, but it is produced naturally in the human gut by daidzein, a major isoflavone predominantly found in soybean, by intestinal bacteria (Setchell and Clerici, 2010). Equol exhibits stronger anti-oxidant and estrogenic activities than daidzein (Hwang et al., 2003; Kinjo et al., 2004; Turner et al., 2004; Rufer and Kulling, 2006) and has been demonstrated to act as vasorelaxant (Jackman et al., 2007), along with having anti-inflammatory properties (Blay et al., 2010), which have both been observed previously in soy isoflavones (Chacko et al., 2005; Hall et al., 2008). However, only 30–50% of the human population can produce equol (Low et al., 2005; Ozasa et al., 2005; Setchell and Cole, 2006; Hall et al., 2007). This suggests that health effects of functional foods supplemented with (*S*)-equol could be more beneficial than daidzein. Several studies have demonstrated that a diet supplemented with natural (*S*)-equol alleviates menopausal symptoms, such as hot flushes and crow’s feet wrinkles (Aso et al., 2012; Oyama et al., 2012).

**FIGURE 1:**
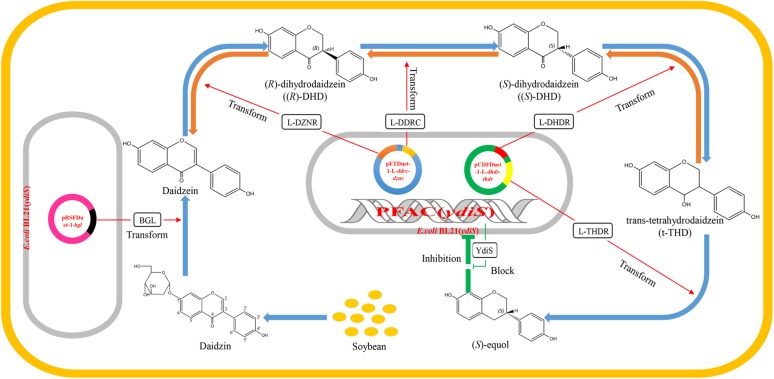
Schematic diagram summarizing the working principle of the engineered equol-producing bacteria. Daidzin is the primary isoflavone that exists in soybean. The first engineered BL21 (*ydiS*) containing *bgl* gene can convert daidzin into daidzein. The fermented liquid was then used as substrate for a second engineered BL21 (*ydiS*) containing equol-producing genes L-*dznr*, L-*ddrc*, L-*dhdr*, and L-*thdr*. The second engineered BL21 (*ydiS*) could then convert daidzein to (*S*)-equol step by step. The engineered BL21 (*ydiS*) could withstand the inhibitory effects of (*S*)-equol by overexpression of a (*S*)-equol resistant gene *ydiS*.

Currently, the majority of equol production is performed by chemical synthesis, although production of (*S*)-equol via bacterial fermentation may have several advantages over chemical synthesis. Specific intestinal bacteria are responsible for the conversion of daidzein to (*S*)-equol, such as *Coriobacteriaceae* sp. and *Lactobacillus* sp. (Setchell and Clerici, 2010). The Hishigaki group has cloned and identified a gene cluster responsible for converting daidzein to (*S*)-equol from an equol-producing strain *Lactococcus* 20–92 (Shimada et al., 2011, 2012). The gene product of L-*dznr* is responsible for converting daidzein into (*R*)-dihydrodaidzein; the L-*ddrc* gene product converts (*R*)-dihydrodaidzein into (*S*)-dihydrodaidzein; the L-*dhdr* gene product converts (*S*)-dihydrodaidzein into *trans*-tetrahydrodaidze; and the L-*thdr* gene product converts *trans*-tetrahydrodaidzein into (*S*)-equol (Shimada et al., 2011, 2012). Lee et al. (2016) constructed a recombinant *Escherichia coli* BL21 strain which can produce (S)-equol *in vitro*. However, Vázquez et al. (2017) reported that isoflavone-derived compounds like (*S*)-equol have the ability to inhibit the growth from many bacteria species. The aim of this study was to obtain an (*S*)-equol resistant host *E. coli*, which can be engineered for (*S*)-equol production by co-expressing the equol-producing genes L-*ddrc*, L-*dznr*, L-*dhdr*, and L-*thdr*. As a result, a putative oxidoreductase gene *ydiS* was identified to be responsible for the (*S*)-equol resistance. An engineered equol-producing bacterial strain was constructed using an (*S*)-equol resistant mutant [*E. coli* BL21 (*ydiS*)] to coexpress the equol-synthesis genes. A two-step method was utilized to convert diadzin to (*S*)-equol under aerobic conditions. All results of this study have been summarized in a schematic diagram (**Figure 1**).

## Materials and Methods

### Chemicals and Reagents

Daidzin, daidzein and (*S*)-equol were purchased from Daicel Chiral Technologies Co., Ltd. (Shanghai, China). The antibiotics and isopropyl-D-thiogalatopyranoside (IPTG) were ordered from Sangon Biotech Bio (Shanghai, China). The restriction enzymes and ligation kit were purchased from TaKaRa Bio (Dalian, China).

### Bacteria Strains, Plasmids, and Growth Conditions

Detailed information regarding the strains and plasmids used in this study is listed in **Supplementary Table 1.** In brief, *E. coli* strains DH5α and BL21 (DE3) were ordered from TaKaRa Bio (Dalian, China) and Transgene Biotech (Beijing, China), respectively. Mariner transposon plasmid pFAC (Wong and Mekalanos, 2000) and a *dap* auxotroph *E. coli* strain WM3064 (Saltikov and Newman, 2003) were obtained from Dr. Gao’s laboratory (Zhejiang University, Hangzhou, China). DH5a *E. coli* (pRK2013) was ordered from Biomedal S. L.^1^. The genes L-*dznr* (GenBank accession number: AB558141.1), L-*ddrc* (GenBank accession number: AB694972.1), L-*dhdr* (GenBank accession number: AB592970.1), L-*thdr* (GenBank accession number: AB592969.1), and *bgl* (GenBank accession number: JQ957567.1) were synthesized and then sub-cloned the pUC57 vector by GenScript Biotechnology (Nanjing, China). The *ydiS* gene was PCR amplified using primers *ydiS*-F: 5-ATG TCG GAT GAC AAA TTT GAT GCC A-3, and *ydiS*-R: 5-ATC GCG CCA ACG AGG GAA TTA-3. The *ydiT* gene of BL21 (*ydiS*) was synthesized and sub-cloned into the pRSFDuet-1 vector by GenScript Biotechnology (Nanjing, China). *E. coli* compatible vectors pRSFDuet-1, pETDuet-1, and pCDFDuet-1 were acquired from Merck Millipore (Germany). *E. coli* strains were grown at 37°C in Lennox broth (LB) or LB agar. When required, 50 μg/mL carbenicillin, 50 μg/mL streptomycin, and 15 μg/mL kanamycin were added to the broth or plates. When required, an anaerobic chamber (anaerobic workstation AW 500, Electrotek Ltd., United Kingdom) was employed to minimize oxygen exposure.

### Transposon Mutagenesis Library Screening

In this study, the transposon of the pFAC plasmid, consisting of a transposable element flanked by two inverted repeats of 27 bps (5^′^-aca ggt tgg ctg ata agt ccc cgg tct-3^′^) and a gentamycin resistance cassette in the middle (aacC1: 534 bp) was used. A gene encoding the hyperactive mariner transposase, and a gene encoding β-lactamase (*bla*) were included in this plasmid (Withers et al., 2014). In theory, the promotor of gentamycin (P_Gm_) transferred together with the mariner transposon, causing adjacent genes to be overexpressed or repressed, dependent on the transcript directions for the gentamycin promotor and its downstream genes. Transposon mutagenesis was prepared via conjugation utilizing pFAC plasmid-carrying *E. coli* WM3064 as the donor strain and BL21 (DE3) as the recipient strain. Transfer of plasmids from WM3064 to BL21 (DE3) were performed via tripartite conjugations using the helper plasmid pRK2013. In brief, bacterial *E. coli* WM3064, BL21 (DE3), and DH5α (pRK2013) were incubated in LB media at 37°C overnight, 500 μL of each bacterium was then mixed together in a 2 mL tube. After centrifuging, the bacterial pellet was resuspend using LB media and transferred onto a dry LB plate (supplemented with 2,6-diaminopimelic acid) in three compact droplets. After incubation for ∼6 h, the bacteria were gathered and streaked onto LB plates supplemented with gentamicin (15 μg/mL). Bacterial colonies were then seeded into 96-well plates containing LB media supplemented with 200 μg/mL (*S*)-equol. The OD_600_ was detected using a Model 680 microplate reader (Bio-Rad, United States) and SP-2000UV spectrometer (Shanghai Spectrum Instruments Co., Ltd.). The equol-resistant character of the five clones was further verified using a tube culture method. Chromosomal DNA of these (*S*)-equol resistant mutants was isolated using an OMEGA Genomic DNA Extraction Kit (Omega, United States). Taxa identification was performed using by 16s rRNA sequencing and BLAST analysis. The mutant *E. coli* strain BL21 (*ydiS*) was then selected for full genome sequencing using Illumina Hiseq at Majorbio Bio-Pharm Technology Co., Ltd., Shanghai, China. The draft genome sequence data of BL21 (*ydiS*) has been deposited in NCBI as Accession Number PIYU00000000.

### Detection of Bacterial Growth Rate

For static culture, 30 mL of LB media were added to a 100 mL flask bottle. After autoclaving, bacteria, antibiotics and chemical reagents were then added into the bottle. Bacterial density (OD_600_) was measured every 3 h. For culturing under shaking conditions, a real-time detection instrument Microscreen-16 (Gering Instrument Manufacturing (Tianjin) Co., Ltd., Tianjin, China) was used. 40 mL of LB media were added to the 50 mL measure bottle, and 400 rpm (equivalent to 100 rpm in the general shake incubator) was utilized for stirring. The optical absorption value was measured at 30-min intervals. Optical absorption was detected at OD_850_, and a conversion factor between OD_850_ and OD_600_ was calculated using *E. coli* before the experiment.

### Batch Culture Fermentation for Equol Production

Batch culture fermentations were cultured without shaking at 37°C, under both anaerobic and aerobic conditions. Briefly, a basic growth medium, LB (Qingdao Hope Bio-Technology Co., Ltd., Qingdao, China), was used to assess the utilization of daidzin or daidzein in the engineered equol-producing *E. coli*. For fermentation, bacteria were aliquoted into 50 mL flask bottles containing 20 mL of culture media supplemented with either daidzin or daidzein. Twenty microliters of IPTG (25 mg/mL) was added to each bottle to induce gene expression. Samples then were collected at 48 and 72 h after IPTG induction to detect equol and daidzein via HPLC. For production of (*S*)-equol from daidzin, a two-step fermentation was attempted in this study. For the first step, daidzin was transformed to daidzein using *E. coli* (pRSFDuet-1-*bgl*) under fermentation 72 h. The fermentation liquid was then collected after centrifugal separation, and the upper liquid was used for preparing a new LB media (LB-D). For the second step, DDDT-BL21 (*ydis*) was inoculated into the LB-D media to detect the equol production.

### HPLC Detection

Identification of equol and daidzein was performed using HPLC according to a previously described method with some modification (Decroos et al., 2005). In brief, 1 mL of each sample was extracted three times with 1 mL acidic ether, then the ether fractions were combined, evaporated to dryness and resuspended in 200 μL of methanol and stored at -20°C until analysis. HPLC analysis was performed using a Waters e2695 system. Fifteen microliter aliquots of each sample were injected and separated using a SunFireTM C18 5 μm column (4.6 mm × 205 mm). The temperature was set at 30 ± 2°C and the flow rate was maintained at 0.8 mL/min. Elution was isocratic with a mobile phase consisting of 0.01% formic acid:methanol:acetonitrile (50:20:30). Equol was detected at 205 nm; daidzein at 254 nm. Calibration curves for the quantification of daidzein and equol were constructed using pure standards obtained from Daicel Chiral Technologies Co., Ltd. (Shanghai, China).

### Statistical Analysis

SPSS Software (version 20.0; SPSS Inc., United States) and the Student’s *t*-test was employed in this study. *P* < 0.05 was considered to be statistically significant.

## Results

### Inhibitory Effects of (*S*)-Equol on Host *E. coli* BL21 (DE3)

Previously, (*S*)-equol was shown to inhibit the growth of representative human gut bacteria (Vázquez et al., 2017). However, the inhibitory effects of the fermentation product (*S*)-equol on host bacterial *E. coli* BL21 (DE3) requiring further investigation. In this study, an *E. coli* strain was engineered to coexpress the four equol-producing genes L-*ddrc*, L-*dznr*, L-*dhdr*, and L-*thdr*, which originated from an equol-producing bacterial *Lactococcus* strain 20–92 (**Supplementary Figure 1**). In order to evaluate the equol-producing activity of the engineered *E. coli*, 50 μg/mL (∼200 μM) daidzein was added to LB culturing media and (*S*)-equol production was detected under both anaerobic and aerobic conditions at different time points after IPTG induction. As demonstrated in **Supplementary Figures 2A,D**, the metabolites from the engineered *E. coli* had a similar HPLC peaks to the reference standard for (*S*)-equol. The LC-MS results further verified that the equol peak detected by HPLC had the same molecular weight as the (*S*)-equol reference standard (**Supplementary Figures 2B,C,E,F**). Previous studies have reported that the equol produced by gut bacteria is (*S*)-equol (Setchell et al., 2005), and that the metabolite produced by *Lactococcus* strain 20–92 is (*S*)-equol (Shimada et al., 2011, 2012), the metabolite produced by the engineered *E. coli* was likely to be (*S*)-equol (**Supplementary Figure 3**). However, bacterial growth rates were inhibited during fermentation (**Supplementary Figure 4**); bacterial density decreased 30 and 37% under anaerobic and aerobic conditions, respectively, after IPTG was added as an inducer for 24 h. In addition, there was almost no bacterial growth observed even cultured for 72 h (**Supplementary Figure 4**). In order to clarify which compounds had inhibitory effects on the growth of BL21 (DE3), bacterial plates were prepared using different compounds. The fermentation product (*S*)-equol did inhibit BL21 (DE3) growth both under both anaerobic and aerobic conditions (**Supplementary Figure 5**), however, daidzein did not cause inhibition. In order to further verify the inhibitory effects of daidzein and equol on bacterial growth, static liquid culture and shake culture experiment were done under aerobic conditions at 37°C. **Supplementary Figure 6** illuminated that equol has the ability to inhibit BL21 (DE3) growth which was dependent on the equol concentration. However, daidzein not inhibit growth and may have slightly promoted bacterial growth for both BL21 (DE3) and BL21 (G2) (**Supplementary Figures 6A,B**).

**FIGURE 2:**
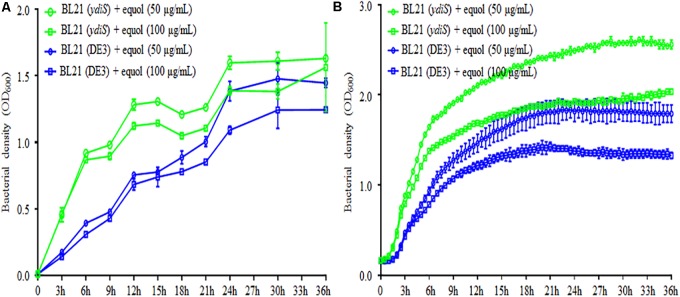
(*S*)-equol resistance of BL21 (DE3) mutant. Growth rates under static **(A)** and shaking **(B)** culture conditions for the BL21 (DE3) and mutant BL21 (*ydiS*) were compared when different concentration of (*S*)-equol were added. At each timepoint, two duplications were measured for each sample, means and standard deviations (SD) were calculated.

### Screening of (*S*)-Equol Resistant BL21 (*ydiS*) Mutants

The feedback inhibitory effects of the fermentation products could prevent high yields of (*S*)-equol product, transposon mutagenesis libraries were constructed and screened in order to develop (*S*)-equol resistant bacteria. In summary, thounds of bacterial clones grown on LB+Gm plates from the mutant library. 93 clones were then randomly picked up and then seeded in a 96-well plate to evaluate their equol resistance. The growth rate was monitored at different time points using a microplate reader. Among screened clones, five clones were identified as growing faster than BL21 (DE3) in the presence of 200 μg/mL (*S*)-equol (data not shown). Twenty-milliliter tubes containing 5 mL of LB and 5 μL of (*S*)-equol were further used to verify the equol-resistance of these clones (**Supplementary Figure 7**). 16s rRNA gene sequencing and BLAST analysis were then used for taxonomy analysis of these clones. Mutant *E. coli* strain BL21 (DE3)_G2 [renamed as BL21 (*ydiS*)] was selected for further verification and study. The bacterial density of BL21 (*ydiS*) was higher than BL21 (DE3) under both static and shaken culture conditions in the presence of 50 or 100 μg/mL (*S*)-equol (**Figure 2**). This BL21 (*ydiS*) strain was then selected for equol production and has been deposited into the China General Microbiological Culture Collection Center (CGMCC No. 14219).

### Identification of Equol Resistant Gene in BL21 (*ydiS*)

**FIGURE 3:**
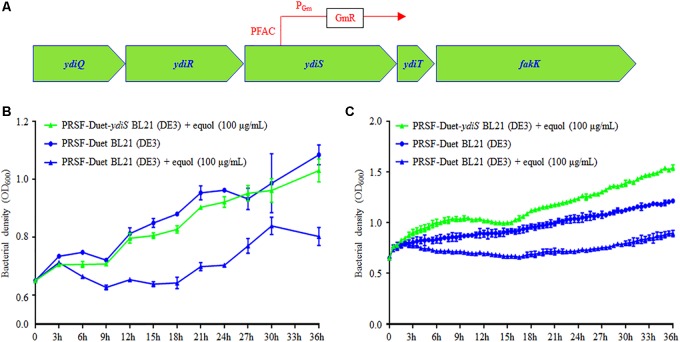
*YdiS* identified as the (*S*)-equol resistant gene in BL21 (*ydiS*). **(A)** Whole genome sequencing revealed that the transposon was inserted at the 307 bp position of the *ydiS* gene; **(B,C)** Overexpression of *ydiS* enabled the *E. coli* BL21 (DE3) to resist (*S*)-equol under static and shaking culture conditions. At each timepoint, two duplications were measured for each sample, means and standard deviations (SD) were calculated.

In order to identify the transposon insert site and infer the equol resistant mechanism of BL21 (*ydiS*), full genome scanning and reverse PCR sequencing were performed. The sequencing results revealed that the PFAC transposon was inserted at 307 bp upstream of the *ydiS* gene. The direction of PFAC Gm promotor is same as the *ydiS* gene (**Figure 3A**). Overexpression of *ydiS* and its downstream gene *ydiT* may have contributed to the equol resistance for BL21 (*ydiS*). *YdiS* and *ydiT* genes were then cloned into pRSFDuet-1, respectively. When the OD_600_ reached ∼0.6, 5 μL of IPTG (25 mg/mL) was then added to induce foreign protein expression under aerobic condition. As indicated in **Figure 3B**, strains that overexpressed *ydiS* had faster growth rates than pRSF-Duet BL21 (DE3) under both static and shaking culture conditions when supplemented with 100 μg/mL (*S*)-equol. However, overexpressed *ydiT* did not growth faster than the pRSF-Duet BL21 (DE3) when 100 μg/mL (*S*)-equol was supplemented (**Supplementary Figure 8**). Together, these results indicated that *ydiS* gene was responsible for the equol resistance in the BL21 (*ydiS*) strain.

### Comparison of the Equol-Producing Activity of BL21 (DE3) and BL21 (*ydiS*)

**FIGURE 4:**
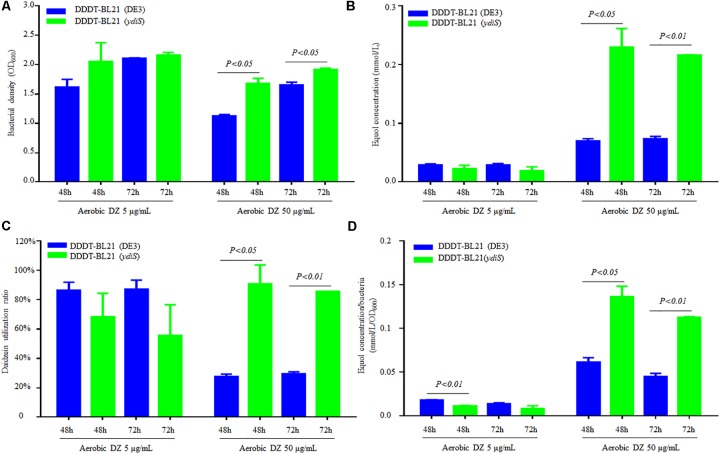
Comparison (*S*)-equol production between DDDT-BL21 (*ydiS*) and DDDT-BL21 (DE3) under aerobic conditions. **(A)** Change in bacterial density; **(B)** Comparison of (*S*)-equol production of DDDT-BL21 (DE3) and DDDT-BL21 (*ydiS*) measured by HPLC; **(C)** Comparison of the daidzein utilization ratio of DDDT-BL21 (DE3) and DDDT-BL21 (*ydiS*); **(D)** Comparison of (*S*)-equol production per bacterium for DDDT-BL21 (DE3) and DDDT-BL21 (*ydiS*). At each timepoint, two duplications were measured for each sample, means and standard deviations (SD) were calculated. The Student’s *t*-test was employed in this study, and *P* < 0.05 was considered to be statistically significant.

No equol production was detected under shaking at 200 rpm (data not shown) and this is consistent with previous reports (Lee et al., 2016). Static culture conditions were utilized to detect the equol production in this study. In order to verify that the mutant strain BL21 (*ydiS*) could grow faster and provide higher yields of (*S*)-equol than BL21 (DE3) during fermentation, the same plasmids pETDuet-1-L-*ddrc*-*dznr* and pCDFDuet-1-L-*dhdr*-*thdr* were transformed into BL21 (DE3) [DDDT-BL21 (DE3)] and BL21 (*ydiS*) [DDDT-BL21 (*ydiS*)], respectively. In 50 mL flasks, 20 mL of LB, carbenicillin (final concentration 50 μg/mL), streptomycin (final concentration 50 μg/mL) and two different concentrations of daidzein (5 μg/mL or 50 μg/mL) were added. Before adding IPTG, the bacterial density was adjusted to OD_600_ = 0.6. After being induced for 48 and 72 h under both anaerobic and aerobic conditions, the bacterial density, daidzein, and equol concentration were measured. The DDDT-BL21 (*ydiS*) was not much better than DDDT-BL21 when 5 μg/mL daidzein was added as the substrate. In contrast, the growth rate, equol yield, and daidzein utilization ratio of DDDT-BL21 (*ydiS*) was greater than DDDT-BL21 (DE3) when 50 μg/mL daidzein was added as substrate both under anaerobic and aerobic conditions (**Figures 4A–C**; **Supplementary Figures 9A–C**). The daidzein utilization ratio of DDDT-BL21 (*ydiS*) reached 90% under aerobic condition after IPTG induction, while the ratio for DDDT-BL21 was only about 27% (**Figure 4C**). In addition, the higher yield of (*S*)-equol for BL21 (*ydiS*) was not only due to faster growth rates, but also because BL21 (*ydiS*) produced more (*S*)-equol than BL21 (DE3) (**Figure 4D** and **Supplementary Figure 9D**).

### Production of (*S*)-Equol Using Daidzin as the Fermentation Substrate

As daidzin is easily collected from soybean meal, using daidzin as fermenting substrate is extremely convenient. As such, a glycoside hydrolysis gene *bgl 1269* (Gang et al., 2012) was cloned into the compatible vector pRSFDuet-1. The pRSFDuet-1-*bgl* plasmid was then transformed into BL21 (*ydiS*) [PRSF-*bgl*- BL21 (*ydiS*)] to verify the gene’s function. After a 72 h induction, daidzin or soybean meal was converted into daidzein under aerobic conditions, with the metabolites from the engineered *E. coli* had a similar HPLC peaks to the reference standard for daidzein (**Supplementary Figure 10**). Interestingly, after transforming pETDuet-1-L-*ddrc*-*dznr*, pCDFDuet-1-L-*dhdr*-*thdr* and pRSFDuet-1-*bgl* into BL21 (*ydiS*), the transformed bacteria could not convert daidzin to (*S*)-equol (data not shown). In order to overcome this hurdle, a two-step method was utilized to transfer daidzin to (*S*)-equol. First, PRSF-*bgl*-BL21(*ydiS*) was used to convert daidzin to daidzein, then the fermentation supernatant was used as substrate for DDDT-BL21 (*ydiS*) to produce (*S*)-equol. After first step fermentation, ∼0.17 mmol/L daidzein was produced from 50 μg/mL daidzin (**Supplementary Figure 11A**). At this point, if the water prepared for LB media was fully replaced with the fermentation supernatant, (*S*)-equol still was not detected after fermentation (data not shown). However, if 10% of the water was replaced with fermentation supernatant when preparing LB media, ∼0.017 mmol/L (*S*)-equol could be detected after the two-step fermentation under both aerobic and anaerobic conditions (**Supplementary Figure 11B**).

## Discussion

Recently, the gut microbiota has become an intensely researched topic (Garrett, 2017). The microbes in the gut have been recognized as important for proper digestive functions, allowing for a variety of dietary components to be metabolized (Koppel et al., 2017), development of the host immune system (Pamer, 2017), as well as for their impacts on some diseases and infections (Boulange et al., 2016). Dietary components, especially polyphenols have been extensively used as functional food components. Previous research has demonstrated that gut microbiota contribute to polyphenol metabolism and affect its bioavailability (Stevens and Maier, 2016); (*S*)-equol and soy isoflavones are typical examples. (*S*)-equol is the metabolite transferred from soybean meal by gut microbes (Setchell and Clerici, 2010), and the equol hypothesis infers that the main functions of soy isoflavones are due the metabolite product (*S*)-equol (Setchell et al., 2002). Furthermore, the safety of (*S*)-equol has been tested (Liu et al., 2016), indicating that further study of the function and molecular mechanism of (*S*)-equol is important. Heemstra et al. (2006) have developed chemical methods to synthesize (*S*)-equol *in vitro*, but natural (*S*)-equol obtained by microbial fermentation is more attractive, especially as it is produced *in vivo*. Considering the vital function of (*S*)-equol, its production could be important for broad applications. In this study, we constructed an engineered *E. coli* mutant BL21 (*ydiS*) that could convert higher concentration of daidzein to (*S*)-equol under aerobic conditions (**Figure 4**). As daidzin is the main form that is found in soybean meal, (*S*)-equol production from daidzin was attempted by coexpressing the genes *bgl*, L-*ddrc*, L-*dznr*, L-*dhdr*, and L-*thdr*, ultimately producing (*S*)-equol using only a two-step method (**Supplementary Figure 11**). Coexpression of all five genes in a single system was not sufficient to convert daidzin to (*S*)-equol (data not shown). This phenomenon could have several explanations: (1) glucose generated by *bgl* conversion may inhibit the enzymatic activity. Not only various microbial β-glucosidases reported previously are strongly inhibited by glucose [11–13], intracellular alpha-L-rhamnosidase activity from *Pseudoalteromonas* sp. also affected by the monosaccharides concentration [8]. In addition, during the two-step fermentation equol could be detected 10% but not when it was fully replaced with fermentation supernatant further supporting this hypothesis. (2) host cells were too old to overexpress other genes after converting daidzin to daidzein. (3) the DDRC, DZNR, DHDR and THDR enzymes were deactivitated during the conversion of daidzin to daidzein. Regardless, the fermentation parameters and process need further adjustment.

Polyphenols have inhibitory activity on bacterial growth (Vázquez et al., 2017), which presents challenges when utilizing high yield fermentation to obtain polyphenol products (Chouhan et al., 2017). Although many antibiotic resistance genes have been identified (Liu and Pop, 2009), few studies have investigated polyphenol resistance. In this study, an (*S*)-equol resistant mutant was generated through a transposon mutagenesis screen. Sequencing and overexpression results revealed that *ydiS*, a putative oxidoreductase gene, was responsible for (*S*)-equol resistance (**Figure 3**). Although Bayer et al. reported that complex I NADH oxidoreductase gene (snoD) in *Staphylococcus aureus* affected the susceptibility of thrombin-induced platelet microbicidal protein 1 (Bayer et al., 2006), the equol resistance mechanism of the putative oxidoreductase *ydiS* gene requires further investigation. The inhibitory effects of (*S*)-equol on bacterial growth may be due to its antioxidant function, as the potential oxidoreductase *ydiS* gene product may be counteract redox active of (*S*)-equol, thereby granting equol resistance. Schrettl et al. (2010) have reported similar phenomenon, which they hypothesize that the primary mechanism of gliotoxin inhibits *Aspergillus fumigatus* growth may be via antioxidant activity. Gliotoxin exposure up-regulates several antioxidant-related proteins and elevates superoxide dismutase activity. Moreover, reactive oxygen species production also increases after exposure to gliotoxin. However, glutathione (GSH) levels were significantly elevated in *Aspergillus nidulans* Δ*gliT* compared to wild-type (Carberry et al., 2012). The *gliT* gene encoded a gliotoxin oxidoreductase exhibits a gliotoxin reductase activity, and overexpression of GliT confers protection against exogenous gliotoxin in *A. nidulans* and *Saccharomyces cerevisiae* (Schrettl et al., 2010).

In summary, a putative oxidoreductase gene *ydiS* was identified to be responsible for (*S*)-equol resistance. As a result, an engineered equol-producing bacterial strain was constructed using an (*S*)-equol resistant mutant [*E. coli* BL21 (*ydiS*)] to coexpress the equol-synthesis genes. A two-step method was constructed to convert diadzin to (*S*)-equol under aerobic conditions, providing a new method for (*S*)-equol fermentation and production. In addition, the method used in this study may be useful for screening resistant host cells as an alternative method for production of anti-bacterial components, such as antibiotics and antibacterial peptides. Recently, herbal medicinal remedies have been gaining increased attention, often being combined with probiotics for therapeutic care. However, the inhibitory effects of herbs on probiotics may prevent their application; therefore, screening for polyphenol resistance genes and probiotics engineering could be beneficial for their combined use.

## Author Contributions

YY and XW conceived and designed the experiments. YY, HL, SM, and HC performed the experiments. YY, HL, LZ, and WL analyzed the data. YY, HL, and XW wrote the paper.

## Conflict of Interest Statement

The authors declare that the research was conducted in the absence of any commercial or financial relationships that could be construed as a potential conflict of interest.

## Footnotes

1 http://lifescience.biomedal.com/
