# Supplementary material for: To Construct an Engineered (S)-Equol Resistant E. coli for in Vitro (S)-Equol Production
Source: Front Microbiol. 2018 Jun 4;9:1182. doi: 10.3389/fmicb.2018.01182 (PMC5994542; doi:10.3389/fmicb.2018.01182)

Supplementary materials

To construct an engineered (*S*)-equol resistant *E. coli* for *in vitro* (*S*)-equol production

Hailiang Li[1, 2, 3, #], Shaoming Mao[3, #], Huahai Chen[1, 2], Liying Zhu[2], wei Liu[2], Xin Wang[2, *] , Yeshi Yin[1, 2, *]

[1]Key Laboratory of Comprehensive Utilization of Advantage Plants Resources in Hunan South, College of Chemistry and Bioengineering, Hunan University of Science and Engineering, Yongzhou, Hunan, China

[2]State Key Laboratory of Breeding Base for Zhejiang Sustainable Pest and Disease Control, Institute of Plant Protection and Microbiology, Zhejiang Academy of Agricultural Sciences, Hangzhou, Zhejiang, China

[3]Hunan Provincial Key Laboratory of Forestry Bitechnology, College of Life Sciences and Technology, Central South University of Forestry and Technology, Changsha, Hunan, China

*** Correspondence:** Yeshi Yin: yinyeshi@126.com; Xin Wang: xxww101@sina.com

[#]These authors contributed equally to this work,

Supplementary Table 1. Strains and plasmids used in this study

Strain and plasmid	Phenotype and genotype	Source or reference
E. coli BL21 (DE3)	F- *ompT hsdS*(*rB$^-$ mB$^-$*) *gal dcm*(DE3)	Transgene Biotech
E. coli DH5α	F-, φ 80d*lacZ* ΔM15, Δ(*lacZYA -argF*)U169, *deoR* , *recA1* , *endA1* , *hsdR17* (*rK$^-$*, *mK$^+$*), *phoA*,	TaKaRa Bio
E. coli WM3064	Δ*dapA*	Saltikov and Newman, 2003
E. coli BL21 (*ydiS*)	Equol-resistant, overexpression of *ydiS*, Gm^R, BL21 (DE3)	This study
pFAC	Mini-himarI mariner transposon with a selectable marker Gm^R	Wong and Mekalanos, 2000
pRK2013	Km^R Tra Mob ColE1	Biomedal S. L.
pUC57	Ap^R; clone vector	Genscript Biotechnology
pUC57-L-*dznr*	*dznr* from Lactococcus 20-92 in Bgl II/Kpn I	This study
pUC57-L-*ddrc*	*ddrc* from Lactococcus 20-92 in BamH I/Not I	This study
pUC57-L-*dhdr*	*dhdr* from Lactococcus 20-92 in BamH I/Not I	This study
pUC57-L-*thdr*	*thdr* from Lactococcus 20-92 in Bgl II/Kpn I	This study
pETDuet-1	Ap^R; T7 promoter-1; T7 promoter-2	Merck Millipore
pCDFDuet-1	Sm^R; T7 promoter-1; T7 promoter-2	Merck Millipore
pRSFDuet-1	Kn^R; T7 promoter-1; T7 promoter-2	Merck Millipore
pETDuet-1-L-*ddrc*	*ddrc* from Lactococcus 20-92 in BamH I/Not I	This study
pETDuet-1-L-*ddrc-dznr*	*ddrc* from Lactococcus 20-92 in BamH I/Not I; *dznr* from Lactococcus 20-92 in Bgl II/Kpn I	This study
pCDFDuet-1-L-*dhdr*	*dhdr* from Lactococcus 20-92 in BamH I/Not I	This study
pCDFDuet-1-L-*dhdr-thdr*	*dhdr* from Lactococcus 20-92 in BamH I/Not I; *thdr* from Lactococcus 20-92 in Bgl II/Kpn I	This study
pRSF-Duet-1-*ydiS*	*ydiS* from BL21 (DE3) in KpnI/XhoI	This study
pRSF-Duet-1-*ydiT*	*ydiS* from BL21 (*ydiS*) in KpnI/XhoI	This study
pRSFDuet-1-*bgl*	*bgl* from uncultured bacterium clone in BamHI/HindIII	This study

Supplementary Figure 1. Schematic of the construction of plasmids pETDuet-1-*L-ddrc-dznr* and pCDFDuet-1-*L-dhdr-thdr* for transformation into *E. coli* BL21 (DE3). pETDuet-1 and pCDFDuet-1 were ordered from Merck Millipore (Germany). pUC57-*L-dznr*, pUC57-*L-ddrc*, pUC57-*L-dhdr*, and pUC57-*L-thdr* were synthesized using the (*S*)-equol producing gene cluster from *Lactococcus* sp. strain 20-92 by GenScript USA Inc. (Nanjing, China). The plasmid pETDuet-1-*L-ddrc* was generated by ligating the double digested products of pETDuet-1 and pUC57-*L-ddrc* together. The plasmid pCDFDuet-1-*L-dhdr* was constructed by ligating the double enzyme digested products of pCDFDuet-1 and pUC57-*L-dhdr* together. The plasmid pETDuet-1-*L-ddrc-dznr* was generated by ligating the double enzyme digested products of pETDuet-1-*L-ddrc* and pUC57-*L-dznr* together. The plasmid pCDFDuet-1-*L-dhdr-thdr* was generated by ligating the double enzyme digest products of pCDFDuet-1-*L-dhdr* and pUC57-*L-thdr* together. (*S*)-equol producing bacteria were then generated by transforming the plasmids pETDuet-1-*L-ddrc-dznr* and pCDFDuet-1-*L-dhdr-thdr* into competent BL21(D3) cells.

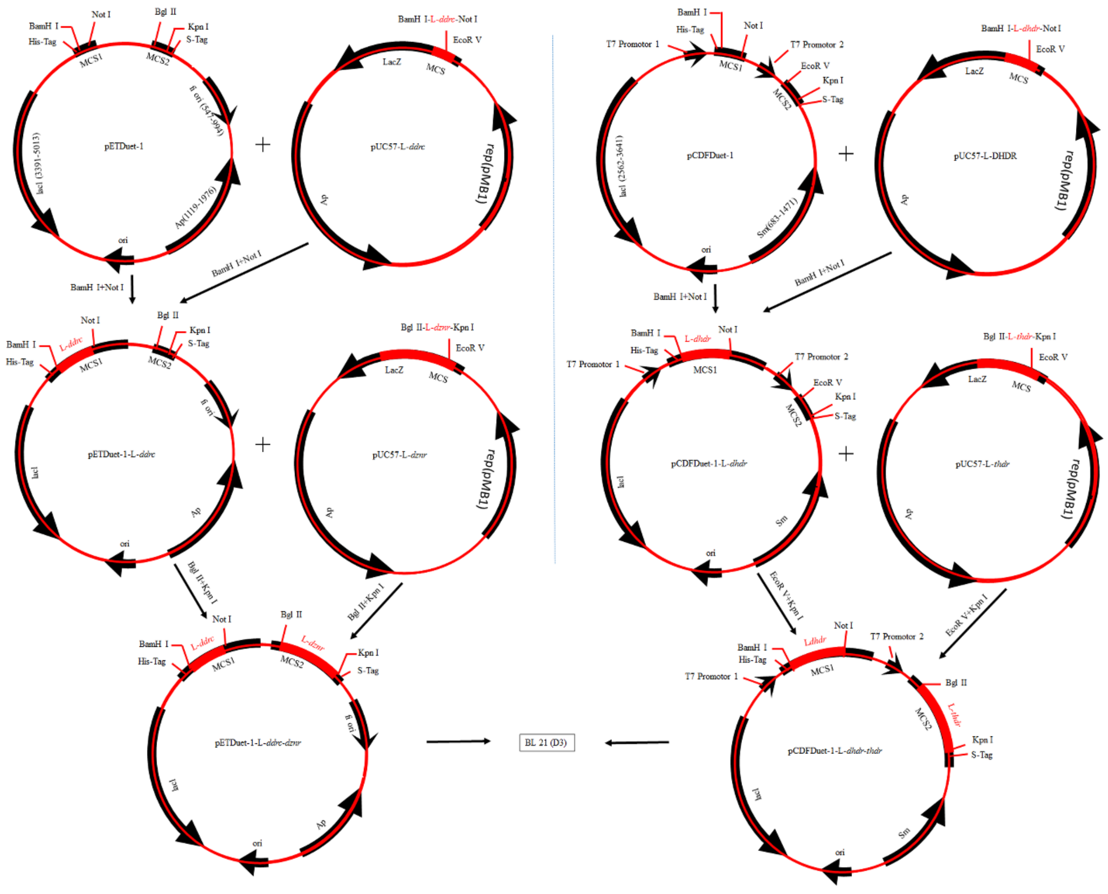

Supplementary Figure 2. Identify the equol production using HPLC and LC-MS/MS. A and D represent the HPLC results using an (*S*)-equol standard and the supernatant from engineered bacteria, respectively. B and E represent the cation exchange MS results using an (*S*)-equol standard and the supernatant from engineered bacteria, respectively. C and F represent the anion exchange MS results using an (*S*)-equol standard and the supernatant from engineered bacteria, respectively. For LC-MS/MS detection, an Agilent 6460 LC/MS (California, USA) equipped with an ESI source was used. Positive and negative modes were used to acquire mass spectra profiles in 100-1000 amu. Operation parameters were: gas temperature, 325 ℃; gas flow, 5 L/min; nebulizer, 45 psi; sheath gas temperature, 350 ℃; sheath gas flow, 11 L/min; capillary voltage, 3000 V (+) and 3500V (-); nozzle voltage, 0 V (+) and 500 V (-); fragmentor, 135 V.

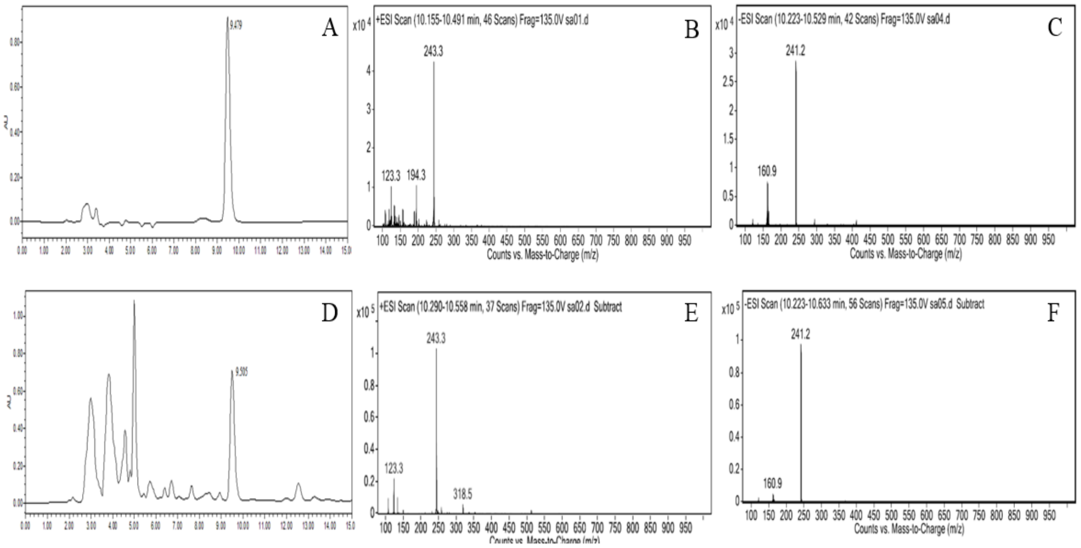

Supplementary Figure 3. Detection of equol chirality using a Chiralcel OJ-H column. A, Reference standard of (*R*)-equol; B, Reference standard of (*S*)-equol; C, Supernatant from engineered bacteria. For chirality detection, an HPLC Shimadzu LC 20 equipped with UV detector SPD-20A was used in this study. Fifteen microliter aliquots of the original sample from fermentation products were injected and separated using a Chiralcel OJ-H column (0.46 cm I.D. × 25 cm L × 5 μm). The temperature was set at 35 °C and the flow rate was maintained at 1.0 mL/min. Elution was isocratic with a mobile phase consisting of hexane/ethanol (70:30). Equol was detected at an absorbance of 220 nm.

A

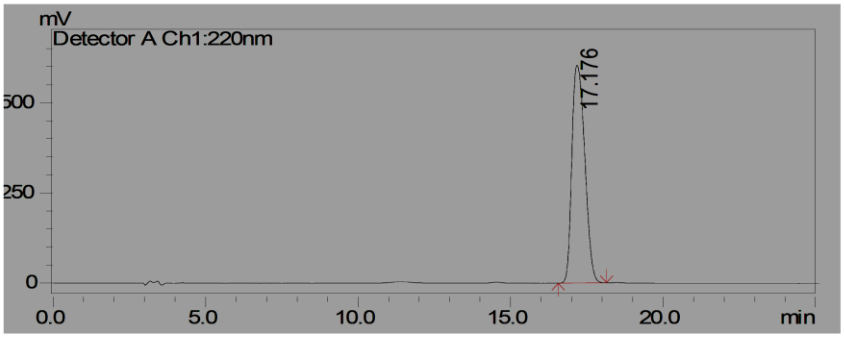

B

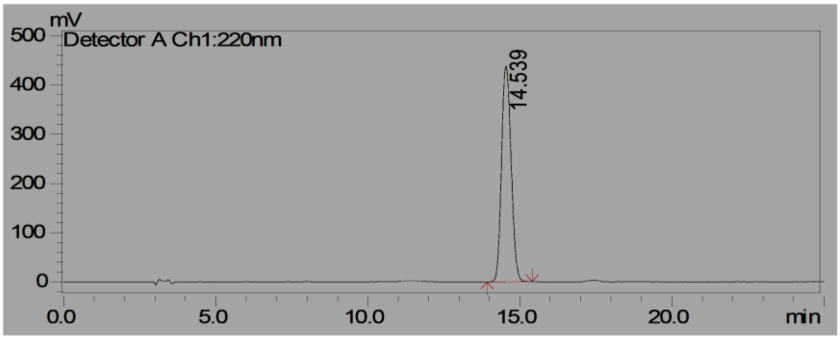

C

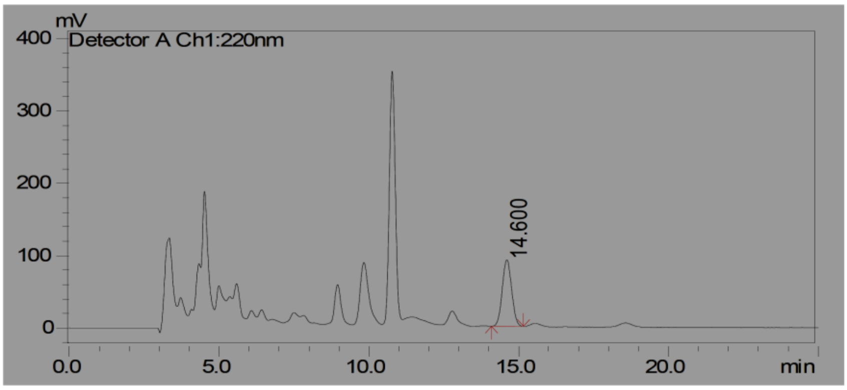

Supplementary Figure 4. Detecting the change in bacterial density of engineered bacteria. For measuring (*S*)-equol production from DDDT-BL21 (DE3), 50 µg/mL daidzein was added as a substrate. Fifty microliters of OD_{600} ~1.0 bacteria were aliquoted into 20 mL cuvettes, each containing 5 mL of culture media. After incubation for 48 h, 5 µL of IPTG (25 mg/mL) was added into each cuvette to induce gene expression. Samples then were collected at 24, 48, 72 and 96 h to measure bacterial density. The bacterial density was measured both before and after IPTG was added. At each timepoint, two duplications were measured for each sample, means and standard deviations (SD) were calculated.

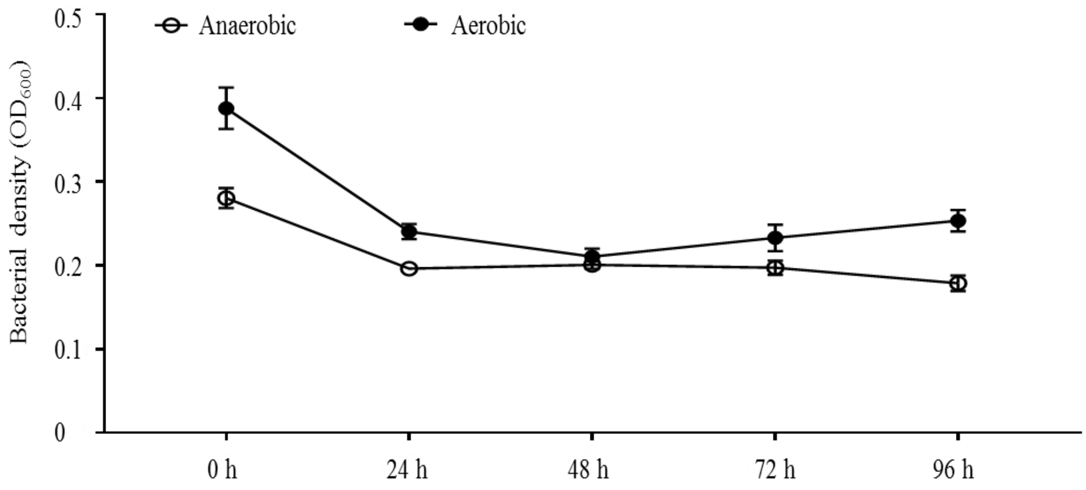

Supplementary Figure 5. Inhibitory effects of (*S*)-equol on *E. coli* BL21 (DE3) under anaerobic and aerobic conditions. A, Inhibitory effect of (*S*)-equol on *E.coli* BL21 (DE3) in an anaerobic incubator; B, Inhibitory effect of (*S*)-equol on *E. coli* BL21 (DE3) in an aerobic incubator. a, five microliters of methanol was added to bacteriostatic paper; b, five microliters of (*S*)-equol (5 mg/mL, diluted in methanol) was added to bacteriostatic paper; c, five microliters of dimethyl sulphoxide (DMSO) was added to bacteriostatic paper; d, five microliters of daidzein (5 mg/mL, diluted using DMSO) was added to bacteriostatic paper.

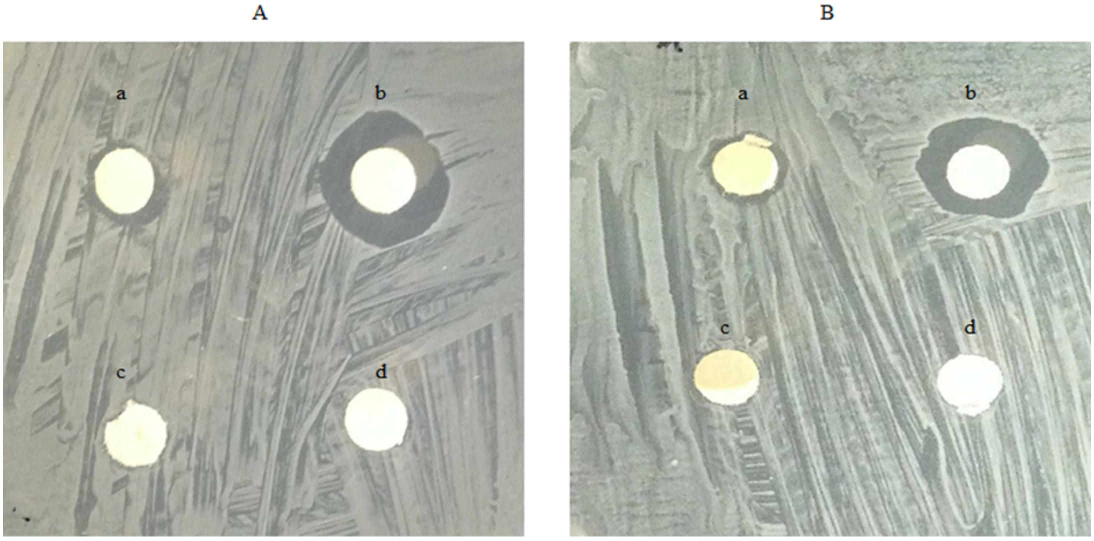

Supplementary Figure 6. Effects of daidzein and (*S*)-equol on *E. coli* aerobic growth under static and shaking conditions. A, Effect of daidzein on *E.coli* growth without shaking; B, Effect of daidzein on *E.coli* growth with shaking; C, Effect of (*S*)-equol on *E. coli* growth without shaking. D, Effect of (*S*)-equol on *E. coli* growth with shaking. At each timepoint, two duplications were measured for each sample, means and standard deviations (SD) were calculated.

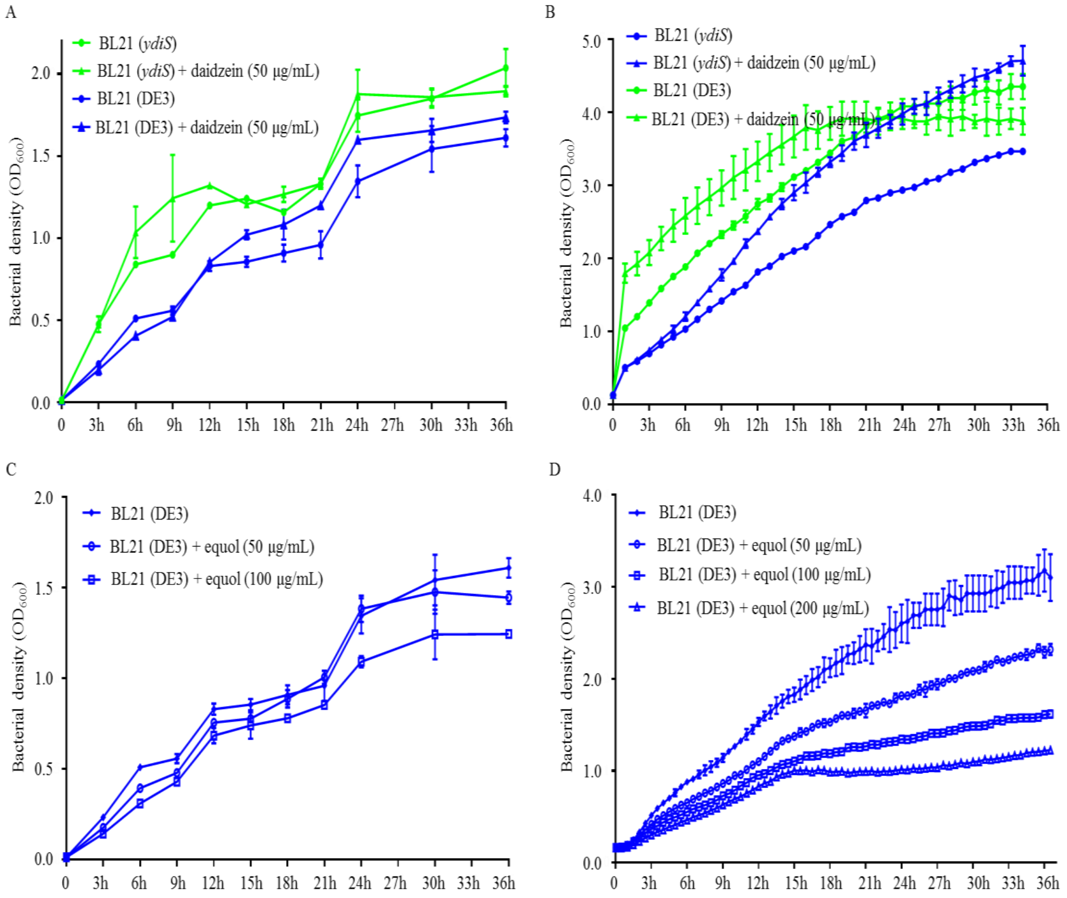

Supplementary Figure 7. Equol resistance verification in screened mutants. Inhibitory effect of (*S*)-equol on *E.coli* BL21 (DE3), where five mutants were detected under static culturing conditions.

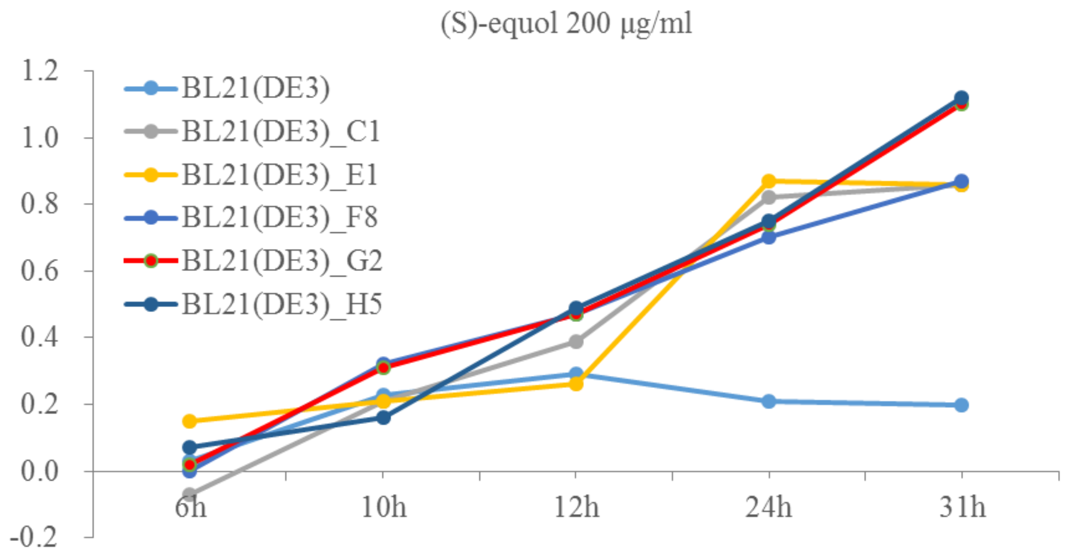

Supplementary Figure 8. Measure (*S*)-equol resistance by YdiT. The bacterial growth rate was compared between empty vector and ydiT overexpression strains under static culture conditions. At each timepoint, two duplications were measured for each sample, means and standard deviations (SD) were calculated.

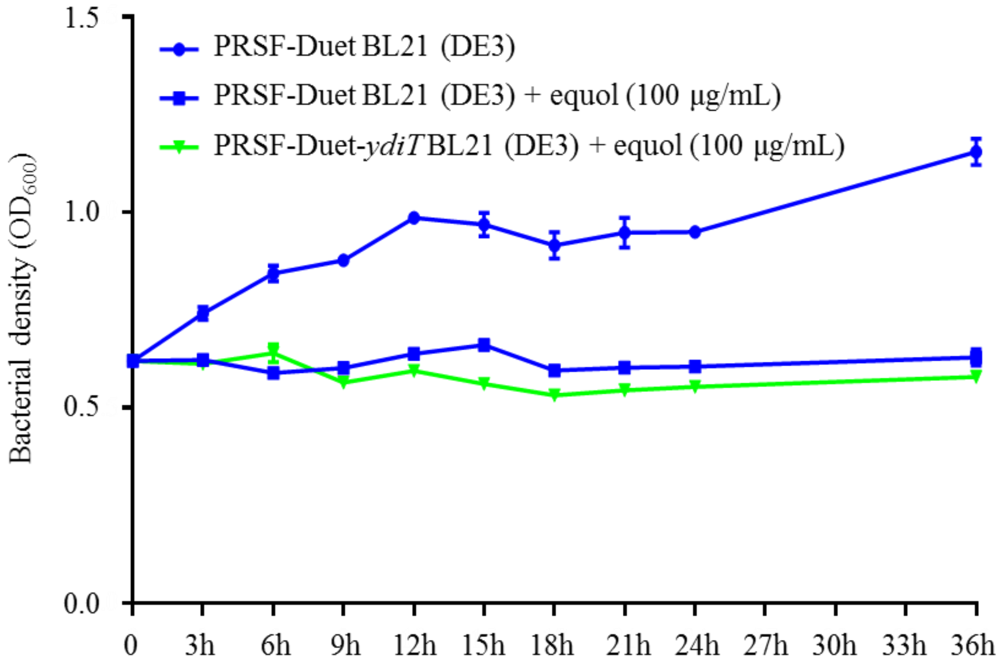

Supplementary Figure 9. Comparison of (*S*)-equol production in DDDT-BL21 (*ydiS*) and DDDT-BL21 (DE3) under anaerobic conditions. A, Change in bacterial density; B, Comparison of (*S*)-equol production from DDDT-BL21 (DE3) and DDDT-BL21 (*ydiS*) as measured by HPLC; C, Comparison of the daidzein utilization ratio of DDDT-BL21 (DE3) and DDDT-BL21 (*ydiS*); D, Comparison of (*S*)-equol production per bacterium for DDDT-BL21 (DE3) and DDDT-BL21(*ydiS*). At each timepoint, two duplications were measured for each sample, means and standard deviations (SD) were calculated.

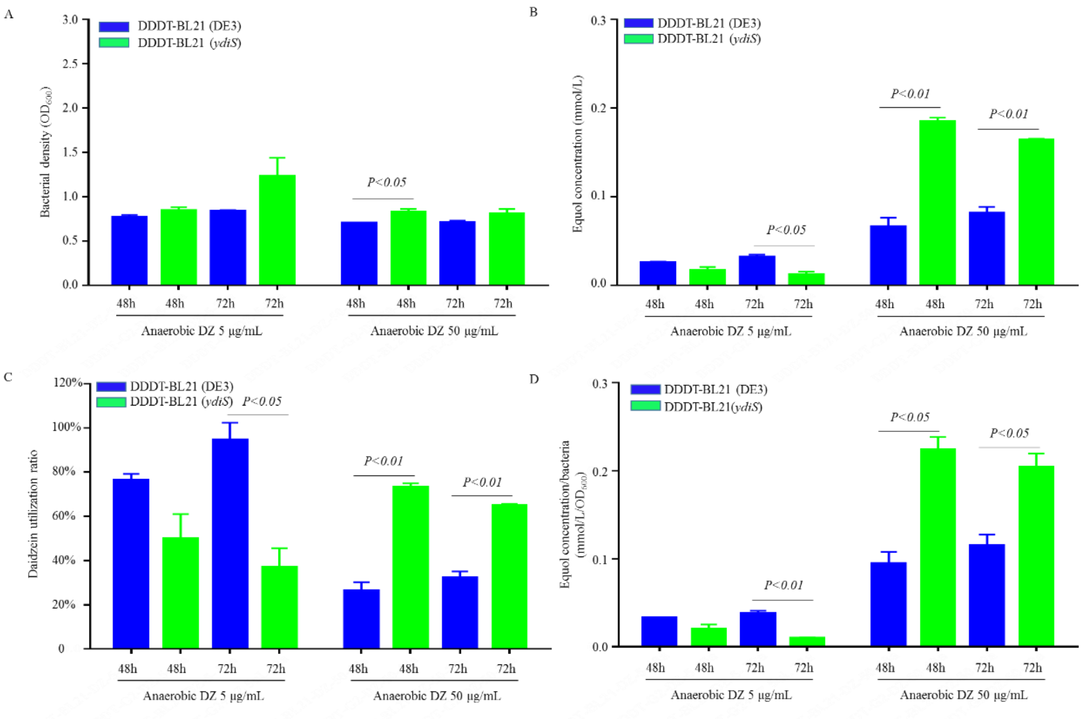

Supplementary Figure 10. Detecting the glycoside-hydrolyzing activity of the *bgl* gene product. A, Standard sample of daidzein. B, Daidzin converted using pRSFDuet-1-*bgl*-BL21 (*ydiS*); C, Soybean meal converted using pRSFDuet-1-*bgl*-BL21 (*ydiS*).

A

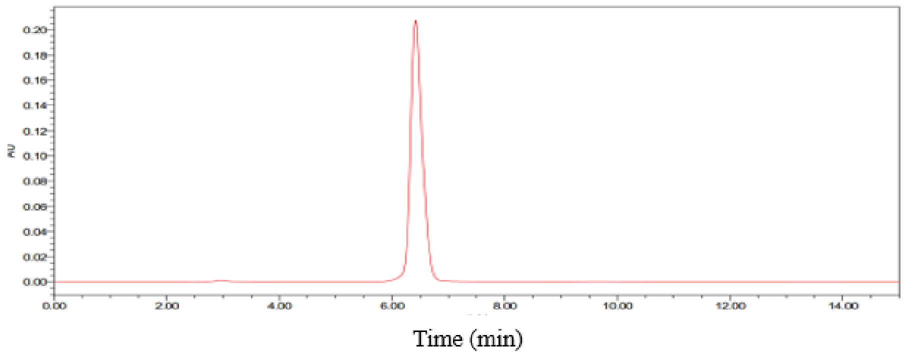

B

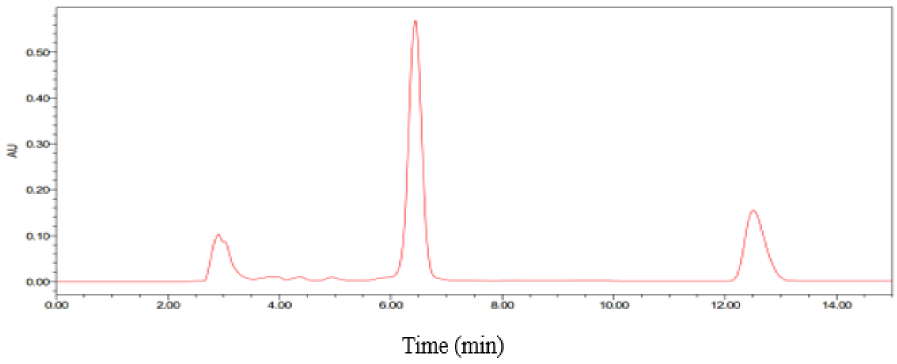

C

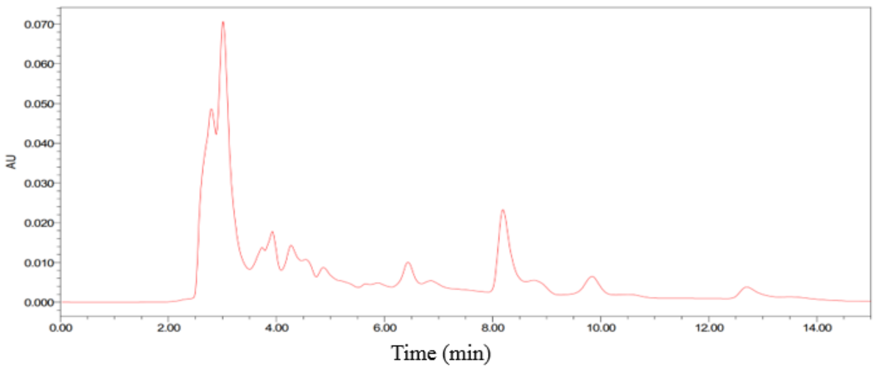

Supplementary Figure 11. Two-step method to produce (*S*)-equol via daidzin fermentation using strains pRSFDuet-1-*bgl*-BL21 (*ydiS*) and DDDT-BL21 (*ydiS*). A, Production of daidzein from daidzin using engineered bacteria pRSFDuet-1-*bgl*-BL21 (*ydiS*). B, Supernatant from A used as a substrate to produce S-equol using engineered bacteria DDDT-BL21 (*ydiS*). Two duplications were measured for each sample, means and standard deviations (SD) were calculated.

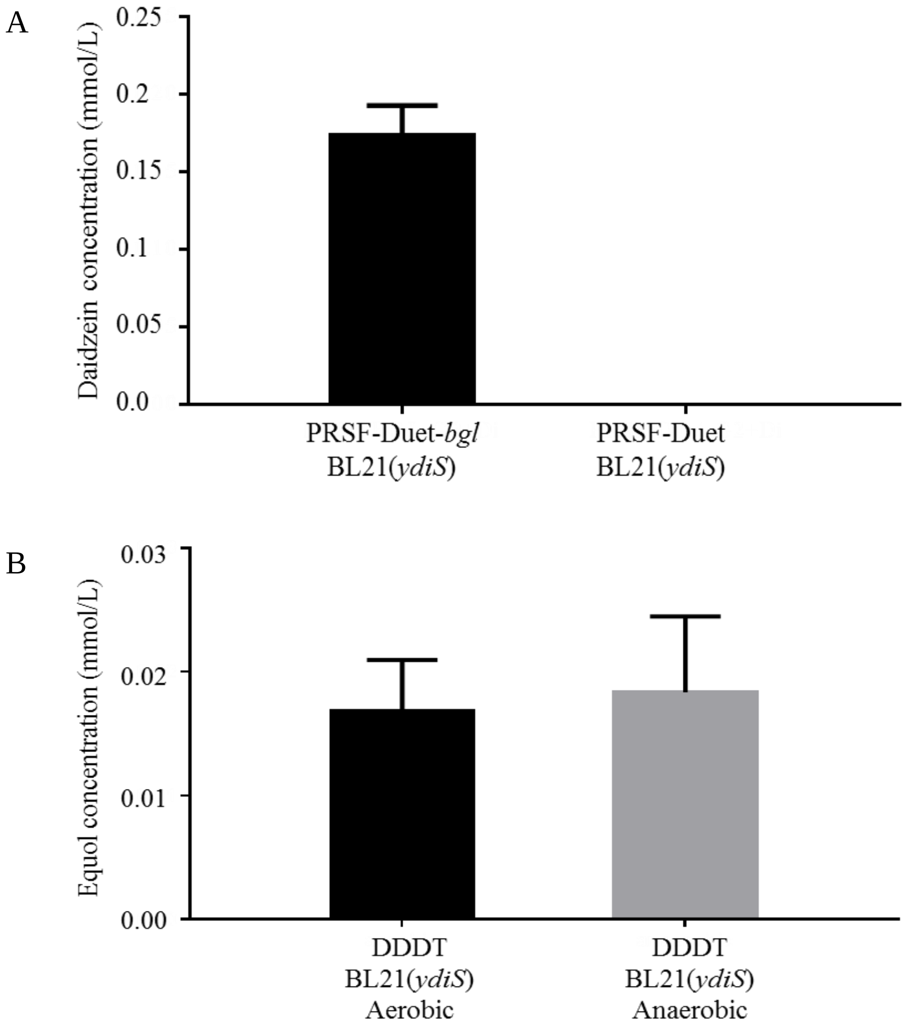

Supplement: Supplementary file 1 [file Presentation_1.pdf]
